# The determinants of the nutritional quality of food provided to the homeless population: a mixed methods systematic review protocol

**DOI:** 10.1186/s13643-023-02286-2

**Published:** 2023-07-10

**Authors:** Divya Ravikumar-Grant, Colette Kelly, Saoirse Nic Gabhainn

**Affiliations:** Health Promotion Department, College of Medicine Nursing and Health Sciences, University of Galway, Galway, Ireland

**Keywords:** Nutrition, Health promotion, Systematic review protocol, Health inequity, Low-income, Public health, Food poverty

## Abstract

**Background:**

Studies assessing the nutritional quality of food provided to the homeless population show deficiencies in micronutrients and excess fat, sugar, and salt. The availability of cheap, energy-dense and nutrient-poor food has changed the profile of people living with homelessness from primarily underweight to obese in western countries. Many factors influence the nutritional quality of food provided to the homeless population such as budget and time constraints, food donations and limited equipment. Nutrient intakes in this population are unlikely to be met outside of charitable meal programmes, making the nutritional quality of these meals crucial. This review will synthesise mixed methods literature with the overarching aim of understanding the determinants of the nutritional quality of food provided to the homeless population.

**Methods:**

This mixed methods systematic review will include English language empirical research studies from Europe, North America and Oceania. The following electronic databases have been chosen for this review: SCOPUS, EMBASE, PsycINFO, EBSCOHost SocIndex and CINAHL. The grey literature databases OpenGrey and ProQuest will also be searched. Quality appraisal will be conducted using the Mixed-Methods Appraisal Tool. Two independent reviewers will be included in study selection, data extraction and quality appraisal. A third reviewer will resolve conflicts. Thematic synthesis will be employed.

**Discussion:**

Results will be organised based on a determinants of health model, to highlight areas where change may be effective, thereby making it more likely to be useful to practitioners and researchers. The iterative steps in the systematic review process will be the focus of this article. Findings from this review will be used to develop best-practice guidelines for stakeholders such as policy makers and service providers to improve the nutritional quality of food provided in the homeless sector.

**Systematic review registration:**

This mixed methods systematic review protocol has been registered with the International Prospective Register of Systematic Reviews (PROSPERO): CRD42021289063.

## Background

There is little evidence examining the nutritional quality of food consumed by the homeless population, despite the body of evidence that suggests that the nutritional needs of this population are not fully met [1–4]. Homelessness often limits access to the basic survival requirement of food, and this has the potential to result in poor nutrition amongst this population [5, 6]. Nutritional quality is described as the value to consumer’s “physical health, growth, development, reproduction, and general well-being” [7], as well as the presence of pathogens, harmful substances, shelf-life and freshness [8]. Studies assessing the nutritional quality of food provided to the homeless population suggest it does not meet the nutritional requirements of this population [9, 10]. However, studies have reported different aspects of nutritional composition as the source of concern. Meals in American soups kitchens have been reported to contain excess fat and be deficient in calcium, fibre and vitamins A and E [11, 12]. High levels of saturated fat were reported in the UK in meals provided through charitable meal services [13].

A review article by Seale et al. (2016), examined the nutritional quality of food consumed by the homeless population and the nutritional quality of food provided by charitable meal services to people who are homeless. Studies included in this review reported low fruit and vegetable consumption amongst members of the homeless population in the UK [14], Ireland [15] and Germany [16]. However, this review, focused solely on the studies that assessed nutrient intake in individuals experiencing homelessness, rather than studies that examined nutrient intake within charitable food services generally. It also focused on methods used to assess dietary intake within this population [6], rather than the factors that determine the nutritional quality of food provided to this population. The focus of the current mixed methods systematic review will be food provided to the homeless population rather than food consumed by members of the homeless population.

### The determinants of food provided to the homeless population

Food services for people experiencing homelessness can range from food vouchers, soup kitchens, shelters [12, 17] and charitable meals from day centres and churches [2, 13]. The nutritional quality of this food can be influenced by several factors. These determinants include food skills and nutrition knowledge of social service providers, food donations, local policies within charitable organisations, governmental policies and limited resources or budget constraints within charitable organisations.

Within charitable food services, a barrier to revising food menus was staff expectations about service users’ food preferences. Service providers were reluctant to deviate from the status quo in relation to the use of food ingredients and continue to feed clients familiar food [18]. Discussions with service providers regarding healthy meal provision have also been met with apprehension due to an assumption that clients would not tolerate ‘exotic’ flavours and did not favour vegetables [19]. Issues with a perceived lack of food skills amongst chefs within charitable food services have also been reported [20].

Donations from food banks and soup kitchens/runs can also be important food sources for service users and homeless services [21]. However, food donations can also be a barrier to obtaining meals of high nutritional quality within charitable food service organisations [18]. The donation of energy-dense and nutrient-poor food such as leftover pastries, pizzas and desserts from restaurants to homeless shelters has been reported in America [12]. In Australia issues with unsaleable waste food obtained through donations have been highlighted as a potential issue in procuring nutritious food for charitable food services [20]. In the UK, a case study of a food aid organisation found that service providers’ dependence on food donations and desire to ‘make use of everything that comes through the door’ has resulted in stockpiling of goods such as instant noodles and pasta [18]. In order to improve the nutritional quality of food provided to the homeless population, it is prudent to consider stricter policies around food donations which may aid charitable food service organisations in improving the nutritional quality of meals for the homeless population.

Economic determinants can play a major role in determining the nutritional quality of food provided to the homeless population. The availability of government aid can determine the type and quality of food for members of the homeless population. In the UK, emergency aid for food provision is primarily child-focused [22]. For example, the national supermarket scheme is a government initiative that provides families with food vouchers through schools to the value of £15 per eligible child [23]. Other UK services such as the Waste Resources Action Programme have been set up to redistribute excess food from supermarkets to food redistribution charities [24]. City-wide policies can also determine the nutritional quality of food within homeless services. In the US, the Food Bank Central New York which runs under the US Hunger Relief Organisation, Feeding America, instituted a “No Soda and No Candy Policy” in 2004 across New York which has been successful in reducing the amount of sugar-sweetened beverages, cakes, cookies, doughnuts and candy donated to food banks [25, 26]. Restrictions within food banks such as this have been suggested in other countries such as Australia to improve the nutritional health of food charity recipients [20].

Budget constraints and limited resources within charitable food services can result in little capacity for improvements in meal quality or increases in food choice [19]. Moreover, initiatives to improve food quality by teaching food skills to service providers may fail due to limited funding that prevents service providers from implementing their learnings [27]. Recipients of charitable food services have reported issues such as a lack of choice, meal repetition, inability to accommodate food preferences, pre-cooked meals, and recycling of leftovers [20]. Issues such as limited kitchen equipment and food storage facilities are important drivers of food choices [13]. This can be an issue for both food service providers in relation to limiting menu expansion [19], and service users in temporary accommodation who may have limited access to cooking or storage facilities [28].

### Consequences of consuming food with poor nutritional quality

The nutritional quality of food provided to the homeless population is a concern due to the declining metabolic health of this population. Although studies from Canada [29] and Portugal [30] have shown overweight the homeless population to be lower than in the general population, studies from the USA have indicated that obesity in the homeless population is as high as 39% [31–33]. In one Irish study, 90% of the sample exhibited abdominal obesity [34]. Tsai and Rosenheck hypothesised that the high level of obesity in their study sample of adults experiencing homelessness may be related to the hunger obesity paradox [33].

The hunger-obesity or food-security paradox is a state of chronic hunger and obesity and has been well documented in populations from lower socio-economic backgrounds [35–37]. In 2012, Koh et al. studied this paradox in the homeless population. The increased availability of cheap, energy-dense, and nutrient-poor food has changed the stereotype of people living with homelessness from primarily underweight to obese in western countries [31]. With charitable meal services being crucial to members of the homeless population and nutrient intakes not likely to be met outside of charitable meal programmes [3], improvements in nutritional quality of food within these organisations is imperative for the improvement of the nutrition-related health of individuals experiencing homelessness.

### Mapping the results onto a determinants of health model

This review will synthesise literature on the determinants of nutritional quality of food for the homeless population in developed countries. The focus of the review will be on food provided to the homeless population rather than food consumed by members of the homeless population. This is because food services such as homeless shelters and soup kitchens have been reported as the primary source of food for members of the homeless populations [9, 33, 38], and studies assessing the nutrient intake of members of the homeless population are sparse due to the transient population of this cohort. A focus on food provision within these settings in this systematic review will provide a basis upon which nutrition guidelines for people experiencing homelessness and their service providers can be produced.

The determinants of nutritional quality, identified through this systematic review will be mapped onto a determinants of health model. This will help to categorise the available evidence and identify where strong evidence exists. The model chosen is the Dahlgren and Whitehead model, which separates factors into interrelated layers, including individual-level factors that affect a person, their social and community networks, living and working conditions and general socio-economic, cultural and environmental conditions [39]. Dahlgren and Whitehead recently reviewed the impact of this model over the past thirty years [40]. They highlight that one of the most successful applications of this model is in multisectoral work. Consideration of this model prior to implementing health promotion action can provide clarity on the roles of each sector and give each sector ownership to create strategies for reducing health inequalities. This is particularly relevant in the field of diet and homelessness as a diverse range of healthcare and social service providers work within homeless service settings and with people experiencing homelessness.

The overarching aim of this systematic review is to understand of the determinants of the nutritional quality of food provided to the homeless population. The purpose is to fill a research gap and provide useful information for service providers, practitioners and researchers that work with the homeless population. A comprehensive understanding of the factors that contribute to the nutritional quality of food provided to the homeless population can help to inform interventions which aim to improve nutritional quality within these settings.

### Objectives

The objectives of this review are:

1. To examine the determinants of the nutritional quality of food provided to the homeless population.
2. To understand the process through which food arrives to services and is distributed within homeless settings.
3. To inform the work of service providers, managers, and practitioners within homeless service settings.
4. To inform the development of food-based guidelines for service providers in homeless charities.

## Methods/design

This mixed methods systematic review protocol has been registered with the International Prospective Register of Systematic Reviews (PROSPERO): CRD42021289063. This protocol has been written with the guidance from the Preferred Reporting Items for Systematic Reviews and Meta-Analysis Protocols (PRISMA-P) checklist [41].

### Eligibility criteria

#### Inclusion criteria

This review will include quantitative, qualitative and mixed-methods peer-reviewed journal articles that explore the determinants of the nutritional quality of food for members of the homeless population. Studies will be limited to those from Europe, North America and Oceania as these areas have countries that are similar in economic status. Studies which also contain data relating to other food insecure populations outside of the homeless population will be included, provided that data is not merged and data relating to the homeless population can be extracted.

#### Exclusion criteria

Peer-reviewed journal articles related to food safety only will be excluded from this review. Studies that contain data relating to the homeless population where this data is merged with data relating to other populations, will be excluded. Studies that solely focus on a nutritional analysis of food consumed by the homeless population will be excluded.

### Sampling strategy

The SPIDER (Sample, Phenomenon of Interest, Design, Evaluation, Research type) tool is employed below to outline the eligibility criteria for this review as this tool was created as an alternative to the commonly used PICO (Population, Intervention, Comparison, Outcome) method which is more suitable for quantitative reviews [42]. The SPIDER tool was chosen because it is applicable to quantitative, qualitative and mixed-methods research [42]. This tool will be used for study selection, as described in Table 1.

**Table 1:** SPIDER Tool [42]

**Sample size**	Study participants are not specified in this review as the focus of the review is the determinants of nutritional quality of food provided to the homeless population. Information could therefore be provided from multiple sources (e.g. people experiencing homelessness, service providers in charitable food services, volunteers in charitable food services, and other stakeholders that work with people experiencing homelessness)
**Phenomenon of interest**	Studies that explore factors that impact the nutritional quality of food provided to the homeless population and barriers and facilitators to nutritional quality of food within charitable or statutory food services
**Design**	Qualitative studies, for example grounded theory, narrative, thematic analysis, ethnography, case studies and action research studies. Quantitative studies, for example randomised control trials, cohort studies, cross-sectional studies, quasi-experimental studies and case-control studies. Mixed methods studies that combine quantitative and qualitative methods
**Evaluation**	Predefined outcome measures will be avoided in this review as they can result in bias or restrictions during the analysis phase
**Research type**	Quantitative, qualitative or mixed methods studies will be included in this review

### Study design

This systematic review is focused on the determinants of the nutritional quality of food provided to the homeless population. Included studies will be primary research studies using qualitative and quantitative methods for data collection and data analysis. This systematic review will exclude review articles, commentary, opinion, and editorial pieces. However, database searches will not be restricted to primary studies only to allow handsearching of reference lists of review articles, commentary, opinion, and editorial pieces.

### Language

English language studies will be included only.

#### Publication year

There will be no restriction on the time of publication to ensure that a comprehensive search is conducted.

### Searches

#### Electronic sources

The website Ulrichsweb was used to verify the databases which index peer-reviewed journals that contained the highest volume of research pertaining to this subject area. These databases were selected to ensure that studies examining the sociological, psychological, medical and behavioural aspects of the topic were included. The following electronic databases have been chosen for this review: SCOPUS, EMBASE, PsycINFO, EBSCOHost SocIndex and CINAHL. The grey literature databases OpenGrey and ProQuest will also be searched in order to ensure a comprehensive review of the literature. A university librarian was also consulted to confirm these database choices.

#### Search strategy

The search strategy for this study was informed by those used in existing studies which were sourced during the preliminary search and was formed with the aid of a university librarian. Search terms were divided into three concepts related to people who are homeless, nutrition or diet and determinants or influences. A total of thirty-eight search terms were included in this search strategy. The search strategy is outlined in Table 2.

**Table 2:** Search strategy to be applied

**#1**	determinant* OR factor* OR cause* OR influence* OR reason*OR impact* OR effect*
**#2**	nutritio* OR diet* OR food OR nourish* OR eating OR meal*
**#3**	homeless* OR houseless OR vagrant* OR “street people” OR vagabond OR destitut* OR “skid row alcoholic” OR squat* OR “of no fixed abode” OR shelter* OR “food bank” OR “food pantry” OR “soup kitchen” OR “community kitchen” OR”community meal centre” OR “community meal center” OR refuge OR refuges OR “temporary housing” OR “emergency accommodation” OR “couch surfing” OR “sofa surfing” OR couchsurfing OR sofasurfing OR “rough sleeper*”
**#4**	#1 AND #2 AND #3 AND #4

### Data management

Following the implementation of the search strategy on the named databases, the lead reviewer (DR) will export the results to Endnote 20 [43]. This software will be used to identify, confirm, and remove duplicates.

### Selection process

Following de-duplication, identified articles will be uploaded to Rayaan, an online systematic review software package. Title and abstract screening will be piloted by DR. Following the piloting phase, title and abstract screening will be conducted by one reviewer (DR). A second reviewer (MH) will screen 20% of the articles uploaded to Rayaan to ensure uniformity in the approach to screening.

At this stage studies will be separated into three groups: (a) studies that potentially meet the eligibility criteria, (b) studies that do not meet the eligibility or (c) studies where it is unclear whether they do or do not meet the eligibility criteria. Studies in categories a and c will be obtained in full-text format. Full-text screening will be piloted by DR and TJ. This will be piloted using 20% of the full-text articles. Following the piloting phase, all full-text studies will be independently screened by two blinded reviewers (DR and TJ). Conflicts will be resolved by a third reviewer (CK). The inclusion and exclusion process will be detailed in a PRISMA flow diagram [44].

### Data collection process and items

Data will be extracted by two reviewer authors (DR and MH) and stored using a data extraction form generated through Microsoft Excel. A pilot data extraction will be conducted by DR and MH on 20% of the included full-text studies in order to compare data extraction techniques, ensure uniformity and make potential modifications. Key information will be extracted from each study including study title, year of publication, country of origin, author, language, study design, aim, setting, participant characteristics, sample size, sampling methods, analysis methods and demographic data on study participants. Data pertaining to the determinants of the nutritional quality of food provided to the homeless population will be extracted from the results and discussion sections of included studies. Extracted data from qualitative studies will include participant quotes and author interpretations, while data extracted from quantitative studies will include numerical information from surveys, questionnaires and tables. Any conflicts during this study phase will be resolved by a third reviewer (CK).

Preliminary searches during the formulation of the research question for this study indicated that evidence in this area of research would consist primarily of qualitative studies. For this reason, an integrated approach will be used to combine the qualitative and quantitative data. Quantitative data will be qualitized by transforming or converting this data into a form that can be subjected to qualitative analysis [45, 46]. The most common approach to integrating data in this way is through thematic synthesis. The process of qualitizing quantitative data is recommended as coding quantitative data is considered to be less error-prone than the process of attributing numerical values to qualitative data. The process of qualitizing quantitative information involves translating quantitative information into textual descriptions [45, 47].

For example, the Joanna Briggs Institute Manual for Evidence Synthesis 2020 [45] outlines that qualitizing can involve changing quantities into stand-alone statements, as illustrated in a mixed methods systematic review by Holley et al. (2017) [48]. One of the studies in this systematic review examined the factors predicting compliance with health regimens by young people with asthma [49]. For example, the quantitative finding “OR = 56.87, 95% 17.15–88.58” was qualitized into “support from nurses as a significant factor in predicting compliance with health regimens by adolescents with asthma” [48]. Once all data have been standardised into the same format, thematic synthesis will then be employed for this review, under the guidance of Thomas and Harden [50].

### Quality assessment

The Mixed Methods Appraisal Tool (MMAT) will be used for the quality assessment of included studies. This tool was chosen due to its suitability for quantitative, qualitative, and mixed methods primary studies. The MMAT contains five categories (qualitative, quantitative randomised controlled trials, quantitative nonrandomized, quantitative descriptive and mixed-methods studies) each with a set of five questions relevant to the study type [51]. The individual scores for each section will be presented as opposed to an overall score, in order to indicate the specific aspects of studies that may increase the risk of bias (Hong et al., 2019). Appraisal of studies will be piloted by DR and MH using 20% of the included full-text studies. Quality assessment will then be performed by two independent reviewers (DR and MH) with a third reviewer (SNG) to resolve any conflicts that may arise.

### Data synthesis

Data will be stored on NVivo 12 and all the results from included studies will be coded. This will require a three-step process [50]. Step one involves line-by-line coding of the data. Step two involves the organisation or grouping of these codes into associated areas to construct descriptive themes. In step three, the descriptive themes will be compared to refine the relationship between them so as to generate analytical themes. A second reviewer (CK) will then examine these codes and any conflicts will be discussed. If consensus cannot be reached conflicts will be resolved by a third reviewer (SNG). Reviewer 1 and reviewer 2 will then examine these topics and use them to combine and condense the codes and topics into themes. In order to facilitate a health promotion-based approach to this synthesis, themes will be organised under the Dahlgren & Whitehead determinants of health model [39].

## Ethics

As this is a systematic review, ethical approval will not be sought.

## Strengths and limitations of this study

- This mixed methods systematic review will include qualitative, quantitative, and mixed methods studies from any year. This will allow for a comprehensive overview of the literature relating to the determinants of the nutritional quality of food provided to the homeless population.
- The Dahlgren and Whitehead determinants of health model will be used to map the evidence sourced in this systematic review. This model will map the determinants of nutritional quality into spheres of influence (from an individual to a socio-economic level). This will aid policymakers or relevant stakeholders in identifying areas where improvements may be most appropriate.
- English language studies only will be included in this review, therefore excluding any studies in this research field that have been published in other languages.
- This review will only use electronic sources. It will consequently exclude any hardcopies of studies and other research work that have not been uploaded to the internet.

## Discussion and dissemination

This systematic review aims to synthesise literature on the determinants of the nutritional quality of food provided to members of the homeless population. Any deviations from this systematic review protocol will be described and justified in the main systematic review paper.

The purpose of this research is to collate the available evidence on factors that determine the nutritional quality of food provided to members of the homeless population. The diverse range of charitable food services that cater to the homeless population widens the range of factors that can determine the nutritional quality of food provided to the homeless population. Issues such as food donations [21], staffing [18], limited resources and budget constraints can affect the nutritional quality of food in different ways [19].

To date, there is no published systematic review that examines the determinants of the nutritional quality of food provided to the homeless population. This systematic review endeavours to produce research that will aid practitioners, policymakers, and other relevant stakeholders in addressing the nutritional needs of the homeless population and when planning interventions to improve nutritional intake within these settings. Searches will not be limited to studies containing a specific participant group and could therefore include people experiencing homelessness, service providers or volunteers in charitable food services or other stakeholders that work with people experiencing homelessness. This will enable searches to gather articles from several different research fields, including nutrition, health promotion and sociology. This approach aims to cater for the multisectoral nature of these settings and consequently make this review more applicable to the multiple stakeholders that work with the homeless population.

Utilising a health promotion-based approach, results will be organised based on a determinants of health model. This will allow the review to identify areas where change may be most appropriate and will facilitate subsequent knowledge translation activities. The findings from this study will be published in a peer-reviewed, open access journal which will be aimed at public health nutrition and health promotion practitioners and policy makers and those who work closely with the homeless population. Findings from this study will be submitted for presentation at relevant both national and international conferences. Other plans for dissemination include social media campaigns, press releases and workshops with service providers in homeless services.

## Data Availability

Any data extracted during this systematic review process can be provided for review. The extracted data analysed during the current study are available from the corresponding author on request.

## References

[1] Dachner N, Tarasuk V (2002). Homeless, “squeegee kids”: food insecurity and daily survival. Soc Sci Med.

[2] Tarasuk V, Dachner N, Li J (2005). Homeless youth in Toronto are nutritionally vulnerable. J Nutr..

[3] Tse C, Tarasuk V (2008). Nutritional assessment of charitable meal programmes serving homeless people in Toronto. Public Health Nutr..

[4] Bunston T, Breton M (1990). The eating patterns and problems of homeless women. J Womens Health..

[5] Maslow AH (1943). A theory of human motivation. Psychol Rev.

[6] Seale JV, Fallaize R, Lovegrove JA (2016). Nutrition and the homeless: the underestimated challenge. Nutr Res Rev..

[7] Köpke U (2005). Organic foods: do they have a role?. Forum Nutr..

[8] Köpke U, Krämer J, Leifert C. Pre-harvest strategies to ensure the microbiological safety of fruit and vegetables from manure-based production systems. Handbook of Organic Food Safety and Quality. Woodhead Publishing Series in Food Science, Technology and Nutrition: Woodhead Publishing; 2007.

[9] Johnson LJ, McCool AC (2003). Dietary intake and nutritional status of older adult homeless women: a pilot study. J Nutr Elder..

[10] Strasser JA, Damrosch S, Gaines J (1991). Nutrition and the homeless person. J. Community Health Nurs..

[11] Lyles CR, Drago-Ferguson S, Lopez A, Seligman HK (2013). Nutritional assessment of free meal programs in San Francisco. Prev Chronic Dis..

[12] Koh KA, Bharel M, Henderson DC (2016). Nutrition for homeless populations: shelters and soup kitchens as opportunities for intervention. Public Health Nutr..

[13] Sprake EF, Russell JM, Barker ME (2014). Food choice and nutrient intake amongst homeless people. J Hum Nutr Diet..

[14] Rushton CM, Wheeler IE. The dietary intake of homeless males sleeping rough in Central London. Wiley Online Library; 1993.

[15] Hickey C, Downey D. Hungry for Change: Social exclusion, food poverty and homelessness in Dublin A Pilot Research Study. Dublin: Focus Ireland; 2003.

[16] Langnase K, Muller MJ (2001). Nutrition and health in an adult urban homeless population in Germany. Public Health Nutr..

[17] Vidgen HA, Gallegos D (2014). Defining food literacy and its components. Appetite..

[18] Pelham Burn SE, Frost CJ, Russell JM, Barker ME (2014). Improving the nutritional quality of charitable meals for homeless and vulnerable adults. A case study of food provision by a food aid organisation in the UK. Appetite..

[19] Frost CJ, Pelham-Burn SE, Russell JM, Barker ME (2016). Improving the nutritional quality of charitable meals for homeless and vulnerable adults: a mixed method study of two meals services in a large English City. J Hunger Environ Nutr..

[20] Booth S, Begley A, Mackintosh B, Kerr DA, Jancey J, Caraher M (2018). Gratitude, resignation and the desire for dignity: lived experience of food charity recipients and their recommendations for improvement, Perth Western Australia. Public Health Nutr..

[21] Clair A, Fledderjohann J, Lalor D, Loopstra R (2020). The housing situations of food bank users in Great Britain. Soc Policy Soc..

[22] Barker M, Russell J (2020). Feeding the food insecure in Britain: learning from the 2020 COVID-19 crisis. Food Secur..

[23] GOV.UK. National free school meals voucher scheme opens to orders. 2021. Available from: https://www.gov.uk/government/news/national-free-school-meals-voucher-scheme-opens-to-orders.

[24] GOV.UK. Cutting food waste: game-changing fund opens. 2019. Available from: https://www.gov.uk/government/news/cutting-food-waste-game-changing-fund-opens.

[25] Campbell E, Webb K, Ross M. Nutrition Focused Food Banking – A Discussion Paper. Institute of Medicine of the National Academies; 2015.

[26] CWH. The Impact of a “No Soda and No Candy” Donation Policy Executive Summary: University of California at Berkeley The Dr. Robert C and Veronica Atkins Center for Weight and Health. 2009.

[27] Rodriguez J, Applebaum J, Stephenson-Hunter C, Tinio A, Shapiro A (2013). Cooking, Healthy Eating, Fitness and Fun (CHEFFs): Qualitative evaluation of a nutrition education program for children living at urban family homeless shelters. Am J Public Health..

[28] Share M, Hennessy M. Food Access and Nutritional Health among Families in Emergency Homeless Accommodation. Dublin: Focus Ireland; 2017. Report No: 978–1–9997657–3–6.

[29] Lee TC, Hanlon JG, Ben-David J, Booth GL, Cantor WJ, Connelly PW (2005). Risk factors for cardiovascular disease in homeless adults. Circulation..

[30] Oliveira LP, Pereira ML, Azevedo A, Lunet N (2012). Risk factors for cardiovascular disease among the homeless and in the general population of the city of Porto Portugal. Cadernos Saude Publica..

[31] Koh KA, Hoy JS, O’Connell JJ, Montgomery P (2012). The hunger-obesity paradox: obesity in the homeless. J Urban Health Bulle NY Acad Med..

[32] Martins DC, Gorman KS, Miller RJ, Murphy L, Sor S, Martins JC (2015). Assessment of food intake, obesity, and health risk among the homeless in rhode Island. Public Health Nurs..

[33] Tsai J, Rosenheck RA (2013). Obesity among chronically homeless adults: is it a problem?. Public Health Rep..

[34] Scott J, Gavin J, Egan AM, Avalos G, Dennedy MC, Bell M (2013). The prevalence of diabetes, pre-diabetes and the metabolic syndrome in an Irish regional homeless population. QJM..

[35] Dhurandhar EJ (2016). The food-insecurity obesity paradox: a resource scarcity hypothesis. Physiol Behav..

[36] Dinour LM, Bergen D, Yeh MC (2007). The food insecurity-obesity paradox: a review of the literature and the role food stamps may play. J Am Diet Assoc..

[37] Scheier LM (2005). What is the hunger-obesity paradox?. J Am Diet Assoc..

[38] Wiecha JL, Dwyer JT, Dunn-Strohecker M (1991). Nutrition and health services needs among the homeless. Public Health Rep..

[39] Dahlgren G, Whitehead M. Policies and strategies to promote social equity in health. Background document to WHO - Strategy paper for Europe. Stockholm, Sweden: Institute for Futures Studies; 1991.

[40] Dahlgren G, Whitehead M (2021). The Dahlgren-Whitehead model of health determinants: 30 years on and still chasing rainbows. Public health..

[41] Moher D, Shamseer L, Clarke M, Ghersi D, Liberati A, Petticrew M (2015). Preferred reporting items for systematic review and meta-analysis protocols (PRISMA-P) 2015 statement. Syst Rev..

[42] Cooke A, Smith D, Booth A (2012). Beyond PICO: the SPIDER tool for qualitative evidence synthesis. Qual Health Res..

[43] Endnote20. Referencing software program. New York NY. Available from: https://endnote.com/product-details.

[44] Page MJ, McKenzie JE, Bossuyt PM, Boutron I, Hoffmann TC, Mulrow CD (2021). The PRISMA 2020 statement: An updated guideline for reporting systematic reviews. J Clin Epidemiol..

[45] Aromataris E, Munn Z. JBI Manual for Evidence Synthesis. 2020. Available from: https://synthesismanual.jbi.global.

[46] Sandelowski M, Voils CI, Barroso J (2006). Defining and designing mixed research synthesis studies. Res Sch..

[47] The Joanna Briggs Institute (2014). Joanna Briggs Institute reviewers’ manual: 2014 edition / supplement methodology for JBI Mixed methods systematic reviews.

[48] Holley S, Morris R, Knibb R, Latter S, Liossi C, Mitchell F (2017). Barriers and facilitators to asthma self-management in adolescents: a systematic review of qualitative and quantitative studies. Pediatr Pulmonol..

[49] Kyngas H (2000). Nurses’ support: essential factor for the good compliance of adolescents with asthma. Nurs Health Sci.

[50] Thomas J, Harden A (2008). Methods for the thematic synthesis of qualitative research in systematic reviews. BMC Med Res Methodol..

[51] Hong QN, Pluye P, Fàbregues S, Bartlett G, Boardman F, Cargo M, et al. Mixed Methods Appraisal Tool (MMAT), version 2018. Registration of Copyright (#1148552), Canadian Intellectual Property Office, Industry Canada; 2018.

